# Lipidomics reveals divergent root and shoot responses to combined nitrogen deficiency and heat stress

**DOI:** 10.3389/fpls.2026.1910848

**Published:** 2026-09-11

**Authors:** Li Jia, Liping Huang, Guangxin Chen, Qiong Yi, Mu Zhang

**Affiliations:** 1 Institute of Agricultural Resources and Environment, Guangdong Academy of Agricultural Sciences/Key Laboratory of Plant Nutrition and Fertilizer in South Region, Ministry of Agriculture and Rural Affairs, Guangzhou, China; 2 College of Food and Drug, Luoyang Normal University, Luoyang, Henan, China; 3 International Research Center for Environmental Membrane Biology, School of Agricultural and Biological Engineering, Foshan University, Foshan, Guangdong, China

**Keywords:** combined abiotic stress, *Glycine max*, membrane lipid remodeling, organ-specific stress response, oxylipin metabolism

## Abstract

**Introduction:**

Soybean productivity is highly sensitive to heat and insufficient nitrogen supply, stresses that can occur simultaneously. However, organ-specific lipid responses to this stress combination remain poorly resolved.

**Methods:**

We exposed hydroponically grown soybean plants to nitrogen deficiency, heat stress (HS) and their combination and compared physiological, lipidomic and gene-expression responses in roots and leaves.

**Results:**

Combined stress caused the largest reductions in biomass and photosynthetic performance and the highest H₂O₂ and MDA accumulation and relative total abundance of candidate oxylipin features. Untargeted LC–MS lipid profiling coupled with WGCNA revealed distinct organ-specific lipid abundance patterns. HS produced the strongest root lipidomic response, characterized by increased abundances of features putatively annotated as polyunsaturated diacylglycerols, lysophospholipids and a gibberellin A12-related compound. Leaves showed their largest lipidomic shift under combined stress, with decreased abundances of photosynthetic membrane-lipid features, increased linoleic-acid- and digalactosylmonoacylglycerol-related features and divergent changes among jasmonate-related features, including cis-jasmone. RT-qPCR showed higher *PLD1* and lower GA20ox expression in roots under HS. In leaves, *FAD2, LOX2, OPR3* and *MYC2* expression increased under all stress treatments, whereas PLA2 expression increased only under HS and combined stress.

**Discussion:**

These findings reveal pronounced tissue specificity in soybean lipid responses to combined nitrogen deficiency and HS and highlight candidate membrane-lipid- and oxylipin-associated responses for further investigation. However, they do not establish direct root–shoot lipid transport or metabolic coordination.

## 1 Introduction

Soybean (*Glycine max*) is an economically important legume crop and a major global source of vegetable oil, plant protein, animal feed and numerous industrial products (Mishra et al., 2024). Its seeds contain high concentrations of protein and oil, making soybean productivity and seed composition important for food, feed and nutritional security (Dilawari et al., 2022). However, soybean growth, yield and seed quality are highly sensitive to environmental constraints. Heat, drought, salinity and nutrient limitations restrict growth, reproductive development and seed protein and oil accumulation (Staniak et al., 2023). These stresses therefore threaten both soybean productivity and harvested-seed quality.

Climate change is increasing the frequency and duration of heat stress. It can also alter soil nutrient availability and plant nutrient requirements. As a result, crops are increasingly exposed to several stresses simultaneously rather than individually (Zhang and Sonnewald, 2017). Heat stress and inadequate nitrogen supply are two major constraints on soybean production (Bagale, 2021; Jumrani et al., 2024). Heat stress (HS) disrupts leaf performance by compromising thylakoid membrane integrity, inactivating photosystem II complexes, and promoting reactive oxygen species (ROS) accumulation, collectively diminishing photosynthetic capacity (Bamagoos et al., 2021; Prasad et al., 2021; Lee et al., 2024). HS also disrupts reproductive development and limits seed filling (El Sabagh et al., 2020). Nitrogen deficiency decreases chlorophyll formation, protein synthesis and nitrogen assimilation (Song et al., 2019; Wang et al., 2022). Although soybean can obtain nitrogen through symbiotic fixation, this process may not always meet plant demand (Cafaro La Menza et al., 2020). Heat stress can also inhibit root growth, nodulation and nitrogen fixation (Aranjuelo et al., 2015). Soybean may therefore experience heat stress and nitrogen limitation simultaneously. Such combinations can produce responses that differ from those induced by either stress alone. However, current mechanistic understanding is still based mainly on individual-stress studies (Zandalinas and Mittler, 2022).

The co-occurrence of abiotic stresses imposes a substantial agronomic burden, and growth losses may exceed those caused by either stress alone (Shabbir et al., 2022). Nitrogen deficiency can also intensify heat-induced photosynthetic inhibition and growth suppression (Ru et al., 2024), highlighting the critical need for balanced N management under warming scenarios (Ru et al., 2022). One possible basis for this interaction is disruption of carbon-nitrogen balance. The SnRK1 complex senses cellular energy and nutrient status and redirects metabolism under resource limitation (Han et al., 2024). Furthermore, recent perspectives highlight that optimizing carbon partitioning under high temperature is a pivotal lever for enhancing crop thermal resilience (Zhao et al., 2025). Thus, combined heat and nitrogen deficiency may intensify competition for carbon and energy among growth, defense and membrane repair. This competition for resources may alter source-sink relations, membrane repair and lipid-associated stress responses.

Lipids play essential roles in membrane stability, carbon storage and stress signaling. Major lipid groups include glycerophospholipids, galactolipids, triacylglycerols and oxylipins (Sarabia et al., 2020; Xue et al., 2024). Their abundance and composition can be strongly altered by heat and nutrient limitation. In leaves, HS promotes chloroplast membrane remodeling and changes the abundance of photosynthetic galactolipids (Higashi et al., 2018; Xiao et al., 2022). In roots, nitrogen deficiency can alter phospholipid and triacylglycerol metabolism, with consequences for membrane function, carbon storage and root development (Qin et al., 2018; Angkawijaya et al., 2019; Amoah et al., 2025). Heat-induced galactolipid remodeling and PA/DG-associated signaling are therefore established components of individual-stress responses (Hou et al., 2016; Higashi et al., 2018). However, it remains unclear how nitrogen deficiency modifies these responses during simultaneous heat exposure. It is also unknown whether the resulting lipid changes follow similar or contrasting patterns in roots and leaves. Lipid metabolism is closely connected with phytohormone signaling. Oxylipins, including jasmonic acid, connect fatty-acid metabolism with the regulation of stress responses (Wasternack and Song, 2016; Simeoni et al., 2022). This lipid-hormone interaction can influence carbon allocation, growth and cellular defense. Under combined HS and nitrogen deficiency, limited carbon and nitrogen availability may constrain both membrane repair and defense metabolism. However, the extent to which this response differs between roots and leaves remains poorly understood.

Lipids and lipid-derived compounds have been proposed to contribute to communication between plant organs. Fatty acids, phosphatidic acid and oxylipins have been associated with local and systemic stress signaling (Hou et al., 2016). Localized stress in roots or shoots can be accompanied by changes in lipid peroxidation and membrane composition in distal tissues (Takahashi et al., 2020; Mcmillan, 2024). However, parallel changes between organs do not necessarily demonstrate the direct transport of lipid molecules. Tissue-resolved lipid profiling is therefore needed to identify organ-specific responses and candidate signaling processes. Studies that simultaneously compare root and leaf lipid responses to combined heat and nutrient limitation remain scarce (Pascual et al., 2022).

Therefore, this study investigated how nitrogen deficiency, HS and their combination influence lipid remodeling in soybean roots and leaves. Physiological measurements were integrated with untargeted lipid profiling and expression analysis of selected lipid-metabolism-related genes to characterize stress- and organ-specific responses. We further examined the relationships between lipid abundance patterns and changes in plant growth, photosynthetic performance and oxidative damage. We hypothesized that nitrogen deficiency and HS would induce distinct lipid-remodeling patterns and that their combination would produce contrasting responses in roots and leaves. This approach aimed to identify lipid classes and metabolic processes potentially involved in soybean acclimation to combined stress.

## 2 Materials and methods

### 2.1 Plant cultivation and stress treatments

Seeds of soybean (*Glycine max* (L.) Merr.) cv. Zhonghuang 13 were obtained from Shandong Xuhong seed industry Co., Ltd., China. The seeds were surface-sterilized in 5% (v/v) commercial bleach for 10 min and subsequently rinsed five times with double-distilled water. They were germinated on moist filter paper at 25 °C. After three days, seedlings of uniform size were selected and transferred to an aerated hydroponic system. Each culture unit contained six seedlings and was supplied with 2 L of half-strength Hoagland nutrient solution (**Supplementary Table 1**). The nutrient solution was completely replaced every three days.

Following a seven-day establishment period, the plants were subjected to four treatments for 10 days. Control plants received complete half-strength Hoagland solution and were maintained at 27/23 °C during the day/night period. Nitrogen-deficient plants received a nitrogen-free half-strength Hoagland solution (**Supplementary Table 2**) at 27/23 °C. Plants subjected to HS received complete nutrient solution and were maintained at 38/35 °C. For the combined treatment, plants received N-free nutrient solution and were maintained at 38/35 °C. The control and nitrogen-deficiency treatments were maintained in one growth chamber, whereas the HS and combined treatments were placed in a second chamber. Both chambers operated under a 16-h light/8-h dark photoperiod, a photosynthetic photon flux density of 350–400 µmol m^−^² s^−^¹ and relative humidity of 60–75%. Culture units were randomly positioned within each chamber and rearranged every 48 h to reduce positional variation.

Each treatment included six independent hydroponic culture units. A culture unit containing six plants represented one biological replicate and experimental unit. Plants within the same unit were treated as subsamples, and their measurements were averaged to obtain a single replicate-level value. Unless otherwise stated, all physiological, biochemical, lipidomic and gene-expression analyses were based on six biological replicates per treatment (*n* = 6).

For tissue-specific analyses, R1 and L1 represent roots and leaves from the Control treatment, respectively. R2 and L2 represent nitrogen deficiency, R3 and L3 represent HS, and R4 and L4 represent combined stress.

### 2.2 Biomass, gas-exchange and photosynthetic pigment measurements

At harvest, roots and shoots were separated, and their fresh masses were measured immediately using a calibrated analytical balance. The collected tissues were subsequently stored at −80 °C until analysis. Net photosynthetic rate (Pn) and stomatal conductance (g_s_) were measured on the youngest fully expanded leaf between 10:00 and 14:00 using a LI-6800 portable photosynthesis system (LI-COR Biosciences, Lincoln, NE, USA). The chamber was maintained at a photosynthetic photon flux density of 1,000 µmol m^−^² s^−^¹, a reference CO_2_ concentration of 400 µmol mol^−^¹, relative humidity of 60–65%, and a flow rate of 500 µmol s^−^¹. Measurements were recorded after gas-exchange parameters had stabilized. Two plants were measured per container, and three stable readings were recorded per leaf. Chlorophyll and carotenoid contents were determined spectrophotometrically using fresh leaf tissue. Briefly, 0.1 g of fresh leaf tissue was placed in a 15-mL centrifuge tube containing 10 mL of acetone and absolute ethanol (1:1, v/v). The tubes were protected from light and maintained in darkness until the leaf tissue was completely decolorized. Absorbance of the pigment extract was measured at 645 and 663 nm for the determination of chlorophyll a (Chl a) and chlorophyll b (Chl b), respectively, and at 470 nm for total carotenoids. Pigment concentrations were calculated using the equations described by Lichtenthaler and Wellburn (1983). Six independent biological replicates were analyzed per treatment (n = 6).

### 2.3 Assessment of oxidative stress and lipid peroxidation

Oxidative stress was assessed by determining hydrogen peroxide (H_2_O_2_) accumulation and malondialdehyde (MDA) content in roots and leaves. For MDA analysis, 0.5 g of fresh tissue was homogenized in a solution containing 0.5% (w/v) thiobarbituric acid and 20% (w/v) trichloroacetic acid. The homogenate was heated at 90 °C for 45 min, cooled to room temperature and centrifuged at 15,000 × *g* for 20 min. The absorbance of the resulting supernatant was recorded at 532 nm. MDA content was calculated using an extinction coefficient of 155 mM^−^¹ cm^−^¹.

H_2_O_2_ content was measured using a commercial assay kit (Beyotime, Shanghai, China) following the manufacturer’s instructions. Concentrations were determined from the corresponding standard curve, with all absorbance readings falling within the linear detection range. H_2_O_2_ concentrations were presented in µmol g^−^¹ FW. Treatment-to-control ratios were calculated separately for reporting fold changes in the Results. Six independent biological replicates were analyzed for each treatment and tissue (*n* = 6).

### 2.4 Untargeted lipidomic profiling and data analysis

Untargeted lipidomic profiling was conducted independently using six biological replicates for each treatment and tissue. For each sample, 0.5 g of frozen tissue was extracted with 1 mL of methanol/water (3:1, v/v). The tissue was homogenized for 4 min and subjected to three 5-min sonication cycles in an ice–water bath. Extracts were then maintained at −40 °C for 1 h and clarified by centrifugation at 17,400 × *g* for 15 min at 4 °C. The resulting supernatants were transferred to autosampler vials. Equal volumes of all extracts were combined to prepare a pooled quality-control sample, which was analyzed periodically throughout the analytical sequence. Features showing an RSD greater than 30% across the pooled QC injections were removed.

Chromatographic separation was conducted on a Waters ACQUITY I-Class PLUS UPLC equipped with an ACQUITY UPLC HSS T3 column (1.8 μm, 2.1 × 100 mm). The UPLC was coupled to a Waters Xevo G2-XS QToF mass spectrometer. The aqueous mobile phase contained 0.1% formic acid, while the organic phase consisted of acetonitrile containing 0.1% formic acid. Separation was performed at 40 °C using a flow rate of 0.4 mL min^−^¹ and an injection volume of 1 μL. Spectra were collected in both positive- and negative-ion electrospray modes using MS^E acquisition in MassLynx v4.2. Low-energy scans were acquired at 2 V, while the high-energy collision voltage was ramped from 10 to 40 V. The scan time was 0.2 s per spectrum. The capillary voltage was set to 2,000 V in positive mode and −1,500 V in negative mode, with a cone voltage of 30 V. Source and desolvation temperatures were 150 and 500 °C, respectively. Cone-gas and desolvation-gas flows were maintained at 50 and 800 L h^−^¹, respectively.

The raw spectra were processed in Progenesis QI to detect and deconvolute features, correct retention-time variation and align samples. Database annotation considered precursor mass, isotope distribution and fragments obtained from the high-energy MS^E scans. METLIN MS/MS and public spectral libraries were searched using tolerance windows of 100 ppm for precursor ions and 50 ppm for fragment ions. Only matches with a Progenesis QI total score of at least 40 were retained. Because authentic standards were not analyzed, all reported identities were treated as putative and did not meet Level 1 identification confidence. When fatty-acyl positions or double-bond configurations could not be resolved, annotations were reported at the lipid-class or sum-composition level.

Database matches originally assigned as gingerglycolipids A, B and C were interpreted as putative monoacyldigalactosylglycerol features because their proposed structures contained a digalactosylglycerol group esterified with a single fatty-acyl chain. They were therefore reported as putative DGMG 18:3, DGMG 18:2 and DGMG 18:1, respectively. The observed *m/z*, retention time, molecular formula, mass error, MS2 score, total annotation score and confidence level of the principal lipid features are presented in **Supplementary Table 5**.

Feature peak areas were normalized against the total peak area of the corresponding sample. PCA was used to examine sample distribution and analytical reproducibility of the QC samples. PLS-DA was conducted using the ropls package in R. Features were classified as candidate differentially accumulated lipids when they met all three criteria: VIP > 1, absolute log_2_ fold change > 1 and *p* < 0.05. Putatively annotated lipid features were classified using LIPID MAPS and HMDB, followed by mapping to KEGG pathways. Pathway enrichment was assessed using a hypergeometric test.

### 2.5 Calculation of relative total oxylipin abundance

Relative total oxylipin abundance (TOA) was calculated from the untargeted lipidomics dataset. Features classified in the annotation database as eicosanoids or octadecanoids were considered oxylipin-associated features. Unoxygenated fatty acids and features without an oxylipin-related annotation were excluded. After total-peak-area normalization, the normalized peak areas of all retained oxylipin features were summed for each biological sample:


$${\text{TOA}}_{i}=\sum_{j=1}^{m}A_{ij}$$

where *A_ij_* is the normalized peak area of oxylipin feature *j* in biological sample *i*, and *m* is the total number of retained oxylipin features. For graphical presentation, TOA was expressed as fold change relative to the mean TOA of the corresponding tissue-specific Control:


$${\text{TOA fold change}}_{i}=\frac{{\text{TOA}}_{i}}{\text{T}\bar{O}{\text{A}}_{\text{Control},\text{ tissue}}}$$

The TOA metric integrates features derived from both enzymatic pathways primarily the lipoxygenase (LOX), α-dioxygenase, and cytochrome P450 branches and non-enzymatic oxidation of polyunsaturated fatty acids. While lipid peroxidation products (e.g., malondialdehyde, measured separately as MDA) directly indicate oxidative damage, the broader TOA encompasses bioactive oxylipins that function as signaling molecules in stress responses (e.g., jasmonates, green leaf volatiles) alongside their non-enzymatic oxidation derivatives. Thus, TOA provides a global proxy for the activation of oxidative lipid metabolism and oxylipin-mediated signaling networks, complementing the specific damage indicators (MDA, H_2_O_2_) and distinguishing general oxidative injury from regulated metabolic reprogramming.

Six biological replicates were analyzed for each treatment and tissue (*n* = 6). For the WGCNA module–trait correlation analysis, the sample-level TOA value from each biological replicate was used, resulting in 24 samples per tissue across the four treatments. Treatment-mean fold-change values were not used in the correlation analysis.

### 2.6 Weighted correlation network analysis

Separate signed weighted correlation networks were constructed for the root and leaf lipidomic datasets using WGCNA v1.74 in R. Each tissue-specific network included 24 samples representing four treatments with six biological replicates per treatment. Candidate soft-thresholding powers from 1 to 30 were evaluated using the pickSoftThreshold function. Powers of 24 and 17 were selected for the root and leaf networks, respectively, as the lowest powers achieving the predefined scale-free topology fit criterion of *R*^2^ ≥ 0.80. The corresponding scale-free topology fit indices were 0.840 and 0.850. Modules were detected using dynamic tree cutting with a minimum module size of 25 and deepSplit=2. Modules with eigengene dissimilarity below 0.25 were merged. Module eigengenes (MEs) were correlated with physiological traits using Pearson correlation. Network robustness was evaluated by comparing module assignments across nearby soft-thresholding powers using the adjusted Rand index.

### 2.7 RNA isolation and quantitative gene-expression analysis

To assess the expression of genes associated with lipid metabolism and hormone signaling, total RNA was extracted from 0.1 g of root or leaf tissue using RNAiso Plus (Takara, China). RNA extraction was followed by chloroform separation and isopropanol precipitation. The resulting RNA pellets were washed with 75% ethanol, air-dried, and dissolved in RNase-free water. RNA integrity was assessed by 1% agarose-gel electrophoresis, while RNA concentration and purity were determined using a NanoDrop 2000 spectrophotometer (Thermo Fisher Scientific, Wilmington, DE, USA). For cDNA synthesis, 1–2 μg of total RNA was reverse-transcribed using 5× PrimeScript RT Master Mix (Perfect Real Time) with dsDNase according to the manufacturer’s instructions. RT-qPCR was performed using ChamQ Universal SYBR qPCR Master Mix (Vazyme, China) in a final reaction volume of 10 μL containing 5 μL of master mix, 0.5 μL of cDNA, 0.5 μL of primer mixture, and 4 μL of RNase-free water. The amplification program consisted of an initial denaturation at 95 °C for 30 s, followed by 40 cycles of 95 °C for 10 s and 60 °C for 30 s. Melting-curve analysis was performed after amplification to assess primer specificity. *Actin* was used as the reference gene based on previous evaluations of Actin-family genes in soybean under heat and nitrogen stress (Gao et al., 2017; Wan et al., 2017) and relative expression was calculated using the 2-ΔΔCt method (Livak and Schmittgen, 2001). Six biological replicates were analyzed per treatment, with three technical reactions per biological replicate. The primer sequences are given in **Supplementary Table 3**.

### 2.8 Data analysis and visualization

Statistical analyses were conducted using Statistica version 10.0 (StatSoft, USA). The analysis included six independent biological replicates for each treatment (*n* = 6). When multiple technical readings were obtained from the same biological sample, they were averaged before statistical testing. Physiological and RT-qPCR variables were analyzed separately by one-way analysis of variance. Following a significant ANOVA result, treatment means were compared using Fisher’s least significant difference test at *p* < 0.05. Data are presented as means ± standard error. Physiological and gene-expression graphs were prepared using SigmaPlot v16 (Grafiti LLC, USA), while specialized lipidomic visualizations were produced using the software specified for the corresponding analyses.

## 3 Results

### 3.1 Effects of nitrogen deficiency and heat stress on soybean growth and photosynthetic performance

**Figure 1:**
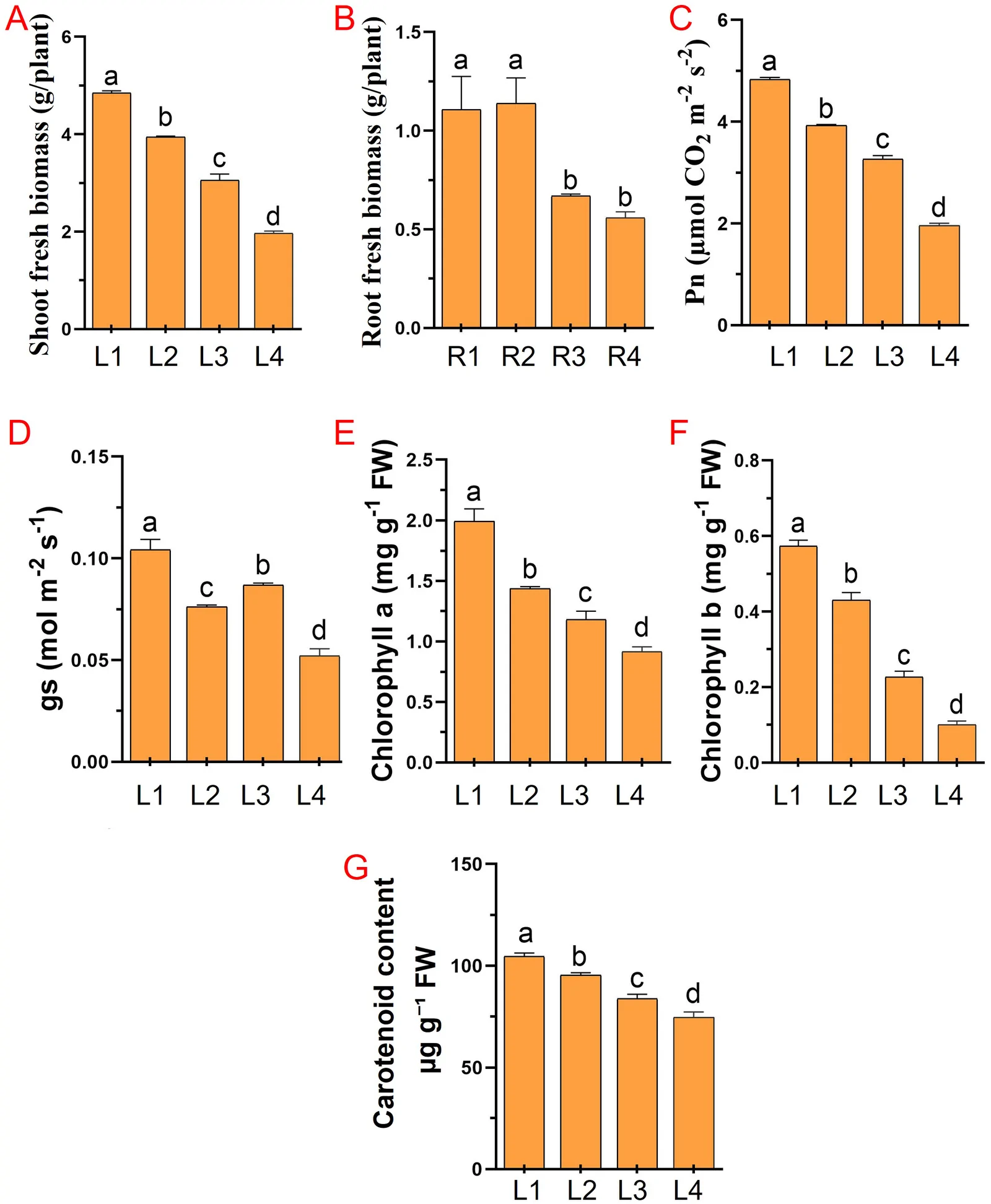
Soybean growth and photosynthetic responses to nitrogen deficiency, heat stress, and their combination. **(A)** shoot fresh biomass, **(B)** root fresh biomass, **(C)** net photosynthetic rate (Pn), **(D)** stomatal conductance (g_s_), **(E)** chlorophyll *a* content, **(F)** chlorophyll *b* content, and **(G)** carotenoid content. R1 and L1 represent root and shoot/leaf measurements under control conditions, respectively; R2 and L2 represent nitrogen deficiency; R3 and L3 represent heat stress (HS); and R4 and L4 represent combined nitrogen deficiency and heat stress. Bars represent means ± SE. Each variable was analyzed separately using one-way analysis of variance (ANOVA), followed by the least significant difference **(LSD)** test. Different lowercase letters indicate significant differences among treatments within each panel at *p* < 0.05.

Soybean showed stress-specific growth reduction, with pronounced effects under combined nitrogen deficiency and heat stress (HS) (**Figure 1**). Nitrogen deficiency (L2, R2) reduced shoot fresh biomass (SFB) by 18.7% but did not significantly affect root fresh biomass (RFB), whereas HS (L3, R3) reduced SFB and RFB by 36.9% and 39.5%, respectively. Combined stress results (L4, R4) in a 58.9% and 48.9% reduction in SFB and RFB, respectively (**Figures 1A, B**). Consistently, for photosynthetic traits, net Pn, and gs were significantly reduced by 54.2% and 50% under combined stress (**Figures 1C, D**), while chlorophyll a (*Chl a*) and chlorophyll b (*Chl b*) contents were decreased by 54.04% and 82.5% under combined stress (**Figures 1E, F**). Carotenoids, another important photosynthesis pigment, were also reduced by 29.2% under combined stress (**Figure 1G**). Among the individual stresses, HS caused significantly greater reductions than nitrogen deficiency in Pn and photosynthetic pigment contents, whereas g_s_ was lower in nitrogen-deficient plants than in HS-treated plants, as indicated by the one-way ANOVA followed by LSD test (**Figures 1C–G**; *p<*0.05).

### 3.2 Oxidative damage and oxylipin accumulation under stress

**Figure 2:**
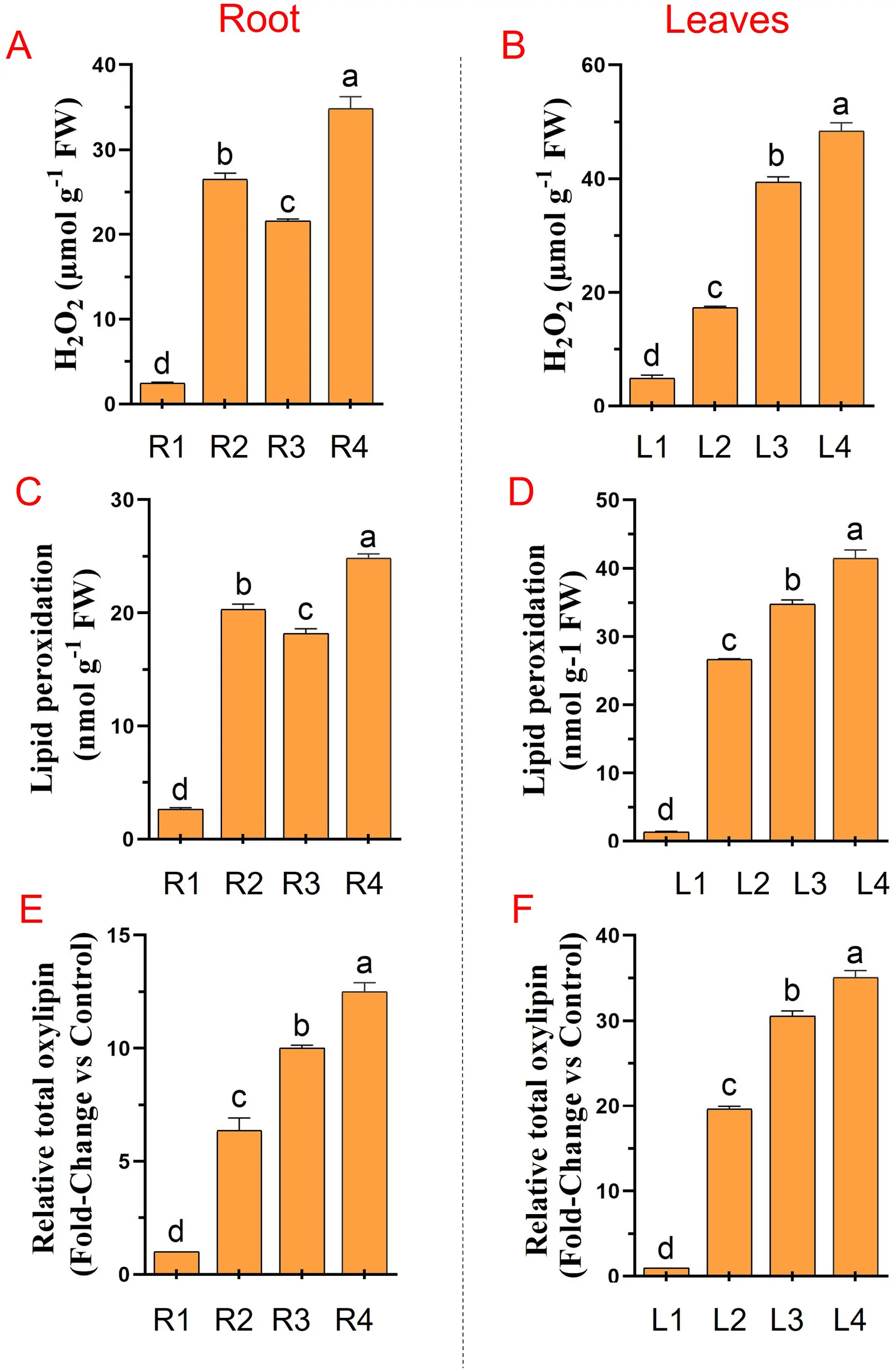
Oxidative damage and oxylipin responses of soybean roots and leaves to nitrogen deficiency, heat stress, and their combination. Hydrogen peroxide (H_2_O_2_) content in **(A)** roots and **(B)** leaves; lipid peroxidation, measured as malondialdehyde (MDA) content, in **(C)** roots and **(D)** leaves; and relative total oxylipin abundance, expressed as fold change relative to the corresponding control, in **(E)** roots and **(F)** leaves. R1 and L1 represent root and leaf measurements under control conditions, respectively; R2 and L2 represent nitrogen deficiency; R3 and L3 represent heat stress (HS); and R4 and L4 represent combined nitrogen deficiency and heat stress. Bars represent means ± SE. Each variable was analyzed separately using one-way analysis of variance (ANOVA), followed by the least significant difference (LSD) test. Different lowercase letters indicate significant differences among treatments within each panel at *p* < 0.05.

Nitrogen deficiency and HS can induce oxidative stress in plants (Luo et al., 2023). In this study, all stress treatments induced significant oxidative damage in soybean, marked by elevated H_2_O_2_ accumulation, and lipid peroxidation (**Figures 2A–D**). Under combined stress, H_2_O_2_ content increased by 13.09-fold in roots and 8.8-fold in leaves relative to their corresponding controls (**Figures 2A, B**; *p* < 0.05). Likewise, lipid peroxidation (based on MDA contents) was significantly increased in roots and leaves compared with the control (**Figures 2C, D**). Oxylipins comprise diverse lipid metabolites generated through both enzymatic pathways, particularly lipoxygenase-mediated reactions, and non-enzymatic oxidation of polyunsaturated fatty acids (Wasternack and Feussner, 2018). Relative total oxylipin abundance (TOA) increased under all stress treatments and was highest under combined stress (**Figures 2E, F**). This increase was interpreted as evidence of altered oxidative lipid metabolism and oxylipin signaling rather than as a direct measure of lipid-peroxidation damage.

### 3.3 Tissue-specific lipidomic responses to stress

**Figure 3:**
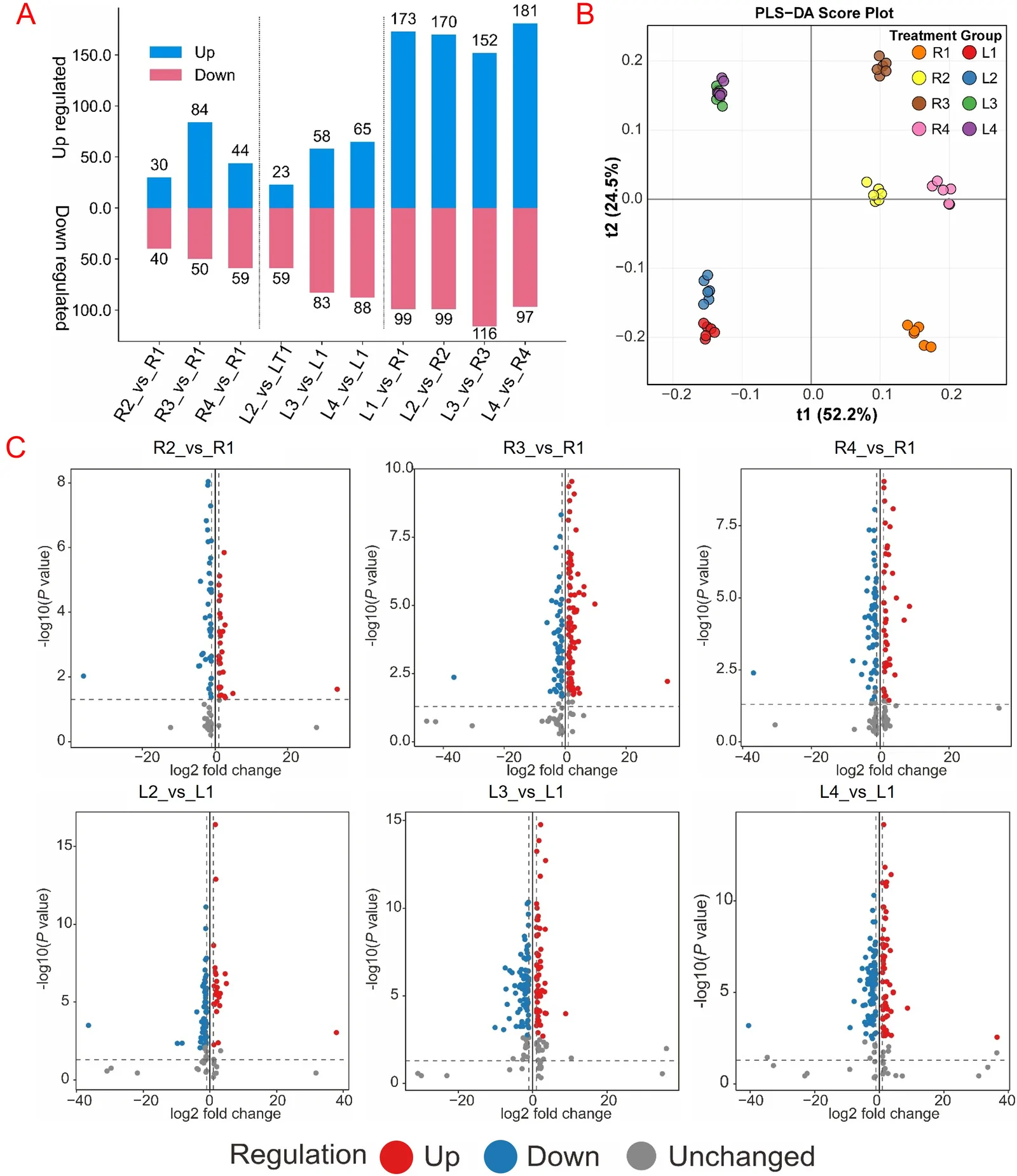
Overview of lipidomic profiles in soybean roots and leaves under different treatments. **(A)** Numbers of differentially accumulated lipids (DALs) identified in roots and leaves across treatment comparisons. **(B)** Partial least-squares discriminant analysis (PLS-DA) score plot showing the separation of lipid profiles among tissues and treatments. **(C)** Volcano plots showing the distribution of upregulated, downregulated, and unchanged lipids in roots and leaves. R1 and L1 represent root and leaf measurements under control conditions, respectively; R2 and L2 represent nitrogen deficiency; R3 and L3 represent heat stress (HS); and R4 and L4 represent combined nitrogen deficiency and heat stress. Within-tissue comparisons indicate each stress treatment relative to its corresponding Control, whereas cross-tissue comparisons indicate leaves relative to roots under the same treatment.

Since both tissues (root and leaves) showed higher lipid peroxidation, lipidomic analysis was performed to examine tissue-specific and stress-specific lipid reprogramming with significant alteration in differentially accumulated lipids (DALs) in soybean under stress (**Figure 3**; **Supplementary Table 4**). Among tissue comparisons, the heat stress (R3_vs_R1) resulted in the most alterations (DALs) in roots (**Figure 3A**; **Supplementary Table 4**). In contrast, in leaves, the combined stress (L4_vs_L1) had the most significant impact, inducing changes in 153 DALs (**Figure 3A**; **Supplementary Table 4**). Cross-tissue comparisons yielded 272, 269, 268 and 278 candidate DAL features for L1_vs_R1, L2_vs_R2, L3_vs_R3 and L4_vs_R4, respectively. In L4_vs_R4, 181 features were higher and 97 were lower in leaves than in roots. Because these comparisons also capture inherent differences between organs, they were interpreted separately from the within-tissue stress responses (**Figure 3A**; **Supplementary Table 4**). The PLS-DA score plot showed a clear separation between treatments and tissues, with a total 76.7% variance for both axes (t1, t2) (**Figure 3B**). Further, the volcano plots of all stress combinations showed diverse regulation of DALs across treatments and tissues (**Figure 3C**).

### 3.4 Classification and overlap of differentially accumulated lipids

**Figure 4:**
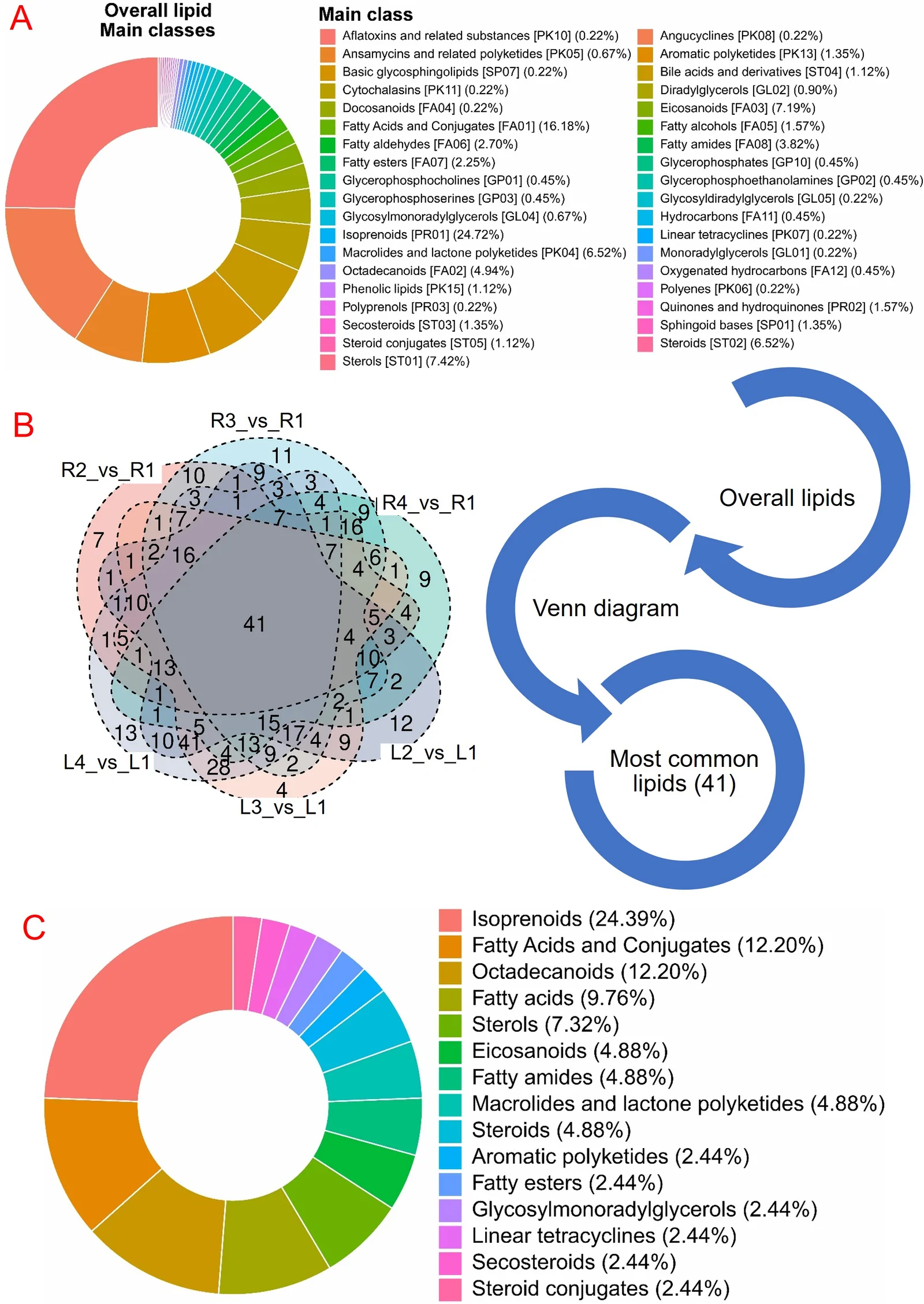
Classification and overlap of differentially accumulated lipids (DALs) in soybean roots and leaves. **(A)** Distribution of the major lipid classes identified across all tissue and treatment comparisons. **(B)** Venn diagram showing the shared and unique DALs among the root and leaf treatment comparisons, including 41 DALs shared across all comparisons. **(C)** Classification of the 41 shared DALs according to their major lipid classes. R1 and L1 represent root and leaf measurements under control conditions, respectively; R2 and L2 represent nitrogen deficiency; R3 and L3 represent heat stress (HS); and R4 and L4 represent combined nitrogen deficiency and heat stress.

Among the candidate DAL features, the most frequently represented database-derived chemical classes were isoprenoids (24.72%), fatty acids and conjugates (16.18%), sterols (7.42%), eicosanoids (7.19%), and macrolides and lactone polyketides (6.52%) (**Figure 4A**). The Venn diagram identified 41 candidate DAL features shared across all six within-tissue stress comparisons (**Figure 4B**). Among these shared features, isoprenoids accounted for the largest proportion (24.39%), followed by fatty acids and conjugates (12.20%) (**Figure 4C**).

### 3.5 Lipid co-abundance networks and their associations with physiological traits

**Figure 5:**
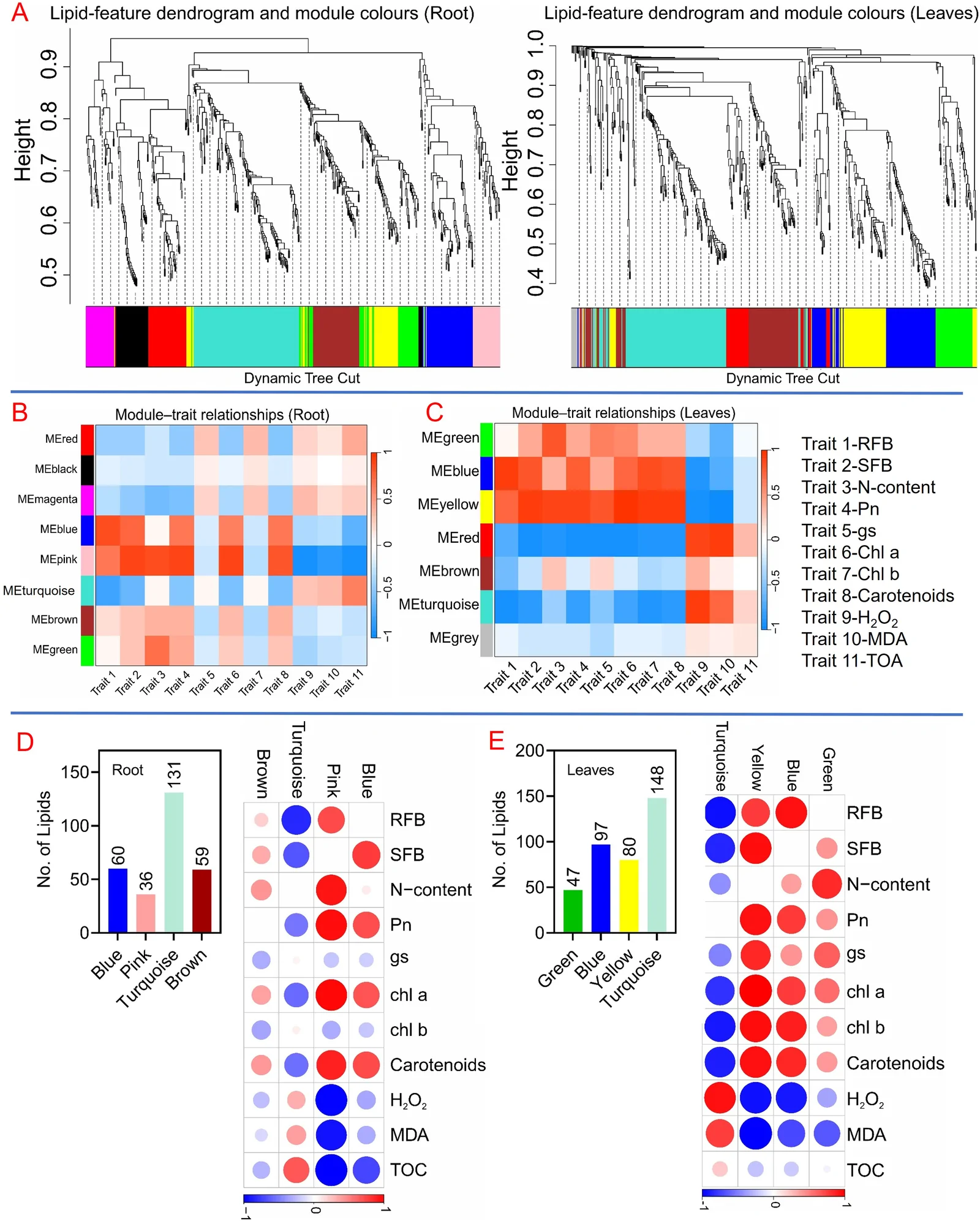
Tissue-specific lipid co-abundance networks and module–trait associations in soybean roots and leaves. **(A)** Hierarchical clustering dendrograms of lipid features in roots and leaves, with module assignments indicated by the dynamic tree-cut colors. **(B, C)** Module–trait relationship heatmaps for roots and leaves, respectively. Trait 1–Trait 11 represent root fresh biomass (RFB), shoot fresh biomass (SFB), N content, net photosynthetic rate (Pn), stomatal conductance (g_s_), chlorophyll *a* (Chl *a*), chlorophyll *b* (Chl *b*), carotenoid content, hydrogen peroxide (H_2_O_2_), malondialdehyde (MDA), and relative total oxylipin abundance (TOA), respectively. **(D, E)** Numbers of lipid features assigned to selected WGCNA modules and their correlations with physiological traits in roots and leaves, respectively. Circle size represents the magnitude of the Pearson correlation coefficient (*r*), while red and blue indicate positive and negative correlations, respectively. Module colors correspond to those identified in the dendrograms.

To examine lipid co-abundance patterns, separate WGCNA networks were constructed for roots and leaves (**Figure 5A**). The analysis grouped lipid features into eight modules in roots (**Figure 5B**) and seven modules in leaves (**Figure 5C**). Among the selected root modules, the turquoise module was the largest, containing 131 lipid features, followed by the blue, brown and pink modules with 60, 59 and 36 features, respectively (**Figure 5D**). In leaves, the turquoise module contained 148 lipid features, followed by the blue, yellow and green modules with 97, 80 and 47 features, respectively (**Figure 5E**).

Module–trait analysis revealed distinct association patterns in roots (**Figures 5B, D**). The blue and pink modules were generally positively correlated with biomass, N content and photosynthetic traits, but negatively correlated with H_2_O_2_, MDA and TOA. The blue module contained features putatively annotated as myristic acid, lysophosphatidylserine and jasmonic-acid-related compounds, whereas the pink module included features putatively annotated as torulene, gibberellin-related compounds and methyl jasmonate. The brown module was positively associated with SFB, N content, Pn and Chl *a* and contained features putatively annotated as linoleic acid, lutein, diacylglycerols and gibberellin A4. In contrast, the turquoise module was generally negatively correlated with growth and photosynthetic traits but positively correlated with H_2_O_2_, MDA and TOA. This module contained features putatively annotated as polyunsaturated diacylglycerols, lysophospholipids, phosphatidylcholine, a DGMG-related feature originally matched to gingerglycolipid B and gibberellin-related compounds (**Figures 5B, D**; **Table 1**).

**Table 1:** Abundance responses of selected WGCNA module-associated lipid features to nitrogen deficiency, heat stress and combined stress in soybean roots and leaves.

ROOTS			
Lipid feature	R2 vs R1	R3 vs R1	R4 vs R1
Blue Module			
Myristic acid	-0.312	-2.302	-0.955
LPS(18:1/0:0)	0.272	-1.820	-0.791
(-)-Jasmonic acid	0.281	-0.891	-1.022
Jasmonic acid	-0.605	0.529	-0.796
Brown Module			
DG (17:2(9Z,12Z)/17:2(9Z,12Z)/0:0)	-0.145	0.271	1.362
9Z,12Z-linoleic acid	0.496	0.288	-0.603
Lutein	-0.968	-0.753	0.828
Gibberellin A4	-0.002	0.564	-0.495
Pink Module			
Torulene	-1.224	-0.744	-1.316
Gibberellin A34	-0.483	-0.756	-0.825
Gibberellin A8	-0.826	-1.007	-1.282
Methyl jasmonate	-1.235	-0.602	-2.463
Turquoise Module			
DG(18:4(6Z,9Z,12Z,15Z)/18:4(6Z,9Z,12Z,15Z)/0:0)	0.704	1.075	0.209
DG(18:3(9Z,12Z,15Z)/18:3(9Z,12Z,15Z)/0:0)	1.684	1.379	1.495
DG(20:5(5Z,8Z,11Z,14Z,17Z)/0:0/20:5(5Z,8Z,11Z,14Z,17Z))	-0.516	0.580	-0.773
Myristoleic acid	-0.146	0.651	0.356
LPA(18:1/0:0)	0.004	0.925	0.721
LPC(0:0/18:1(6Z))	-0.335	1.161	1.130
LPE(16:0/0:0)	-0.588	2.093	0.335
LPE(18:1(9Z)/0:0)	-0.346	0.661	0.473
LPS(18:3(6Z,9Z,12Z)/0:0)	-0.910	-0.053	-0.057
Gingerglycolipid B	1.066	1.312	0.584
Gibberellin A12	0.154	0.983	0.148
Gibberellin A8-catabolite	-0.227	0.382	0.326

Leaves			
Lipid feature	L2 vs L1	L3 vs L1	L4 vs L1
Green Module			
Gibberellin A12	-0.804	-0.483	-0.587
Lutein	0.684	-0.198	-0.740
(-)-Jasmonic acid	-0.615	0.657	0.913
Jasmonic acid	-1.049	-1.215	-1.096
cis-jasmone	-0.217	0.241	0.637
Blue Module			
DG(18:4(6Z,9Z,12Z,15Z)/18:4(6Z,9Z,12Z,15Z)/0:0)	-0.717	-2.577	-2.434
Myristoleic acid	-1.144	0.265	-0.016
Palmitic acid	-0.436	0.671	0.008
myristic acid	-0.386	0.401	0.234
PS(18:3(6Z,9Z,12Z)/0:0)	0.087	-0.996	-0.535
MGDG(20:5(5Z,8Z,11Z,14Z,17Z)/16:3(7Z,10Z,13Z))	-0.112	-3.591	-3.626
Gibberellin A8-catabolite	0.357	0.658	0.472
Gibberellin A29-catabolite	0.705	1.547	1.106
Yellow Module			
DG(20:5(5Z,8Z,11Z,14Z,17Z)/0:0/20:5(5Z,8Z,11Z,14Z,17Z))	-0.147	0.528	-0.626
DG(17:2(9Z,12Z)/17:2(9Z,12Z)/0:0)	0.013	0.718	-0.408
LPA(18:1/0:0)	-0.075	-0.298	-0.369
PC(0:0/18:1(6Z))	-0.256	-0.450	-0.360
LPC(21:0/0:0)	-1.547	-2.425	-2.676
Turquoise			
DG(18:3(9Z,12Z,15Z)/18:3(9Z,12Z,15Z)/0:0)	0.179	0.686	1.307
9Z,12Z-linoleic acid	0.404	3.632	2.822
LPE(16:0/0:0)	-0.278	-0.394	0.280
Gingerglycolipid A	-0.373	1.059	1.171
Gingerglycolipid B	-0.318	2.155	1.053
Torulene	0.223	1.056	0.895
Gibberellin A34	-1.087	-0.768	1.076
Gibberellin A4	-0.232	-0.083	-0.334
α-terpinene	-0.292	-2.203	-1.862
Gibberellin A8	0.053	0.586	0.215
Methyl jasmonate	-1.408	-1.271	-1.300

All compound names represent putative database annotations based on precursor-mass agreement, isotope-pattern similarity and MSE fragment matching. None of these features were confirmed using authentic standards. Values represent log₂ fold changes calculated from normalized peak areas relative to the corresponding tissue-specific control. Positive and negative values indicate increased and decreased normalized abundance, respectively. Supporting annotation evidence and confidence information are provided in **Supplementary Table 5**.

Leaf modules also showed distinct module-specific association patterns (**Figures 5C, E**). The blue and yellow modules were generally positively correlated with growth and photosynthetic traits but negatively correlated with H_2_O_2_, MDA and TOA. The blue module contained features putatively annotated as diacylglycerols, fatty acids, MGDG, phosphatidylserine and gibberellin catabolites. The yellow module included putatively annotated diacylglycerol, LPA, PC and LPC features. The green module was positively associated with several growth and photosynthetic traits and contained features putatively annotated as jasmonate-related compounds, lutein and gibberellin A12. In contrast, the turquoise module was generally negatively correlated with growth and photosynthetic traits but positively correlated with H_2_O_2_ and MDA. It contained features putatively annotated as linoleic acid, DGMG-related features originally matched to gingerglycolipids A and B, carotenoids, gibberellin-related compounds and methyl jasmonate (**Figures 5C, E**; **Table 1**).

### 3.6 Overall tissue-specific lipid reprogramming under treatments

Integration of the lipidomic and WGCNA results revealed treatment-specific lipid-reprogramming patterns in soybean under nitrogen deficiency, heat stress, and their combination (**Table 1**).

In roots, the most pronounced lipid alterations were observed under HS. The turquoise co-abundance module contained several membrane-related lipid features that increased under this treatment, including putative DG 18:3/18:3 (log2FC = 1.379), DG 18:4/18:4 (log2FC = 1.075), LPA 18:1 (database match: PA 18:1/0:0; log2FC = 0.925), LPE 16:0 (database match: PE 16:0/0:0; log2FC = 2.093), and LPC 18:1 (database match: PC 0:0/18:1; log_2_FC = 1.161). Their coordinated abundance patterns were consistent with stress-associated membrane-lipid remodeling in roots. The same module also contained a putatively annotated GA12-related feature (log2FC = 0.983), whose increased relative abundance under HS suggests a possible association between membrane remodeling and growth-related hormone responses. In contrast, a putatively annotated methyl jasmonate feature in the pink module decreased under combined stress (log2FC = −2.463), while jasmonic-acid-related features showed variable responses among treatments. These patterns indicate changes in jasmonate-related metabolism rather than suppression of the entire pathway. Compared with HS, alone nitrogen deficiency (R2 vs R1) produced relatively modest lipidomic changes, whereas under combined stress (R4 vs R1), several components of the HS-associated lipid signature were attenuated but not completely reversed (**Table 1**).

Leaves exhibited their most pronounced lipidomic shift under combined stress. The blue module contained several photosynthetic membrane-lipid features that decreased under the combined treatment, consistent with extensive thylakoid lipid remodeling. The putatively annotated photosynthetic galactolipid MGDG (20:5/16:3) was severely downregulated (log2FC = -3.626), as was the polyunsaturated DG (18:4/18:4/0:0) (log2FC = -2.434). In contrast, the turquoise module contained features showing increased normalized abundances, including 9Z,12Z-linoleic acid under HS (log2FC = 3.632) and combined stress (log2FC = 2.822). Putative DGMG 18:3 and DGMG 18:2 features, originally matched to gingerglycolipids A and B, respectively, also increased. A feature putatively annotated as jasmonic acid decreased under combined stress (log2FC = −1.096), whereas features annotated as (−)-jasmonic acid and cis-jasmone showed positive log2FC values of 0.913 and 0.637, respectively. These steady-state abundance patterns suggest possible changes in oxylipin-related metabolism but do not establish pathway direction or metabolic flux. Compared with the other treatments, nitrogen deficiency (L2 vs L1) induced relatively modest changes, including slight increases in putative DGMG features, originally matched to gingerglycolipids, and lutein. In contrast, individual HS (L3 vs L1) produced a pronounced lipid-remodeling response. The blue module contained decreased putative MGDG 20:5/16:3; log2FC = −3.591 and DG 18:4/18:4; log2FC = −2.577 features. Concurrently, the turquoise module contained an increased 9Z,12Z-linoleic acid feature (log2FC = 3.632), a polyunsaturated fatty acid associated with oxylipin metabolism.

### 3.7 Expression analysis of selected lipid-metabolism- and hormone-signaling-related genes

To characterize gene-expression patterns accompanying the observed lipidomic reprogramming, we quantified the relative expression of selected genes. These included *FAD2*, which is involved in fatty-acid desaturation; *LOX2* and *OPR3*, which participate in jasmonate biosynthesis; *MYC2*, a regulator of jasmonate-responsive gene expression; *GA20ox*, which is involved in gibberellin biosynthesis; and *PLD1* and *PLA2*, which encode phospholipases involved in membrane-lipid metabolism and signaling.

**Figure 6:**
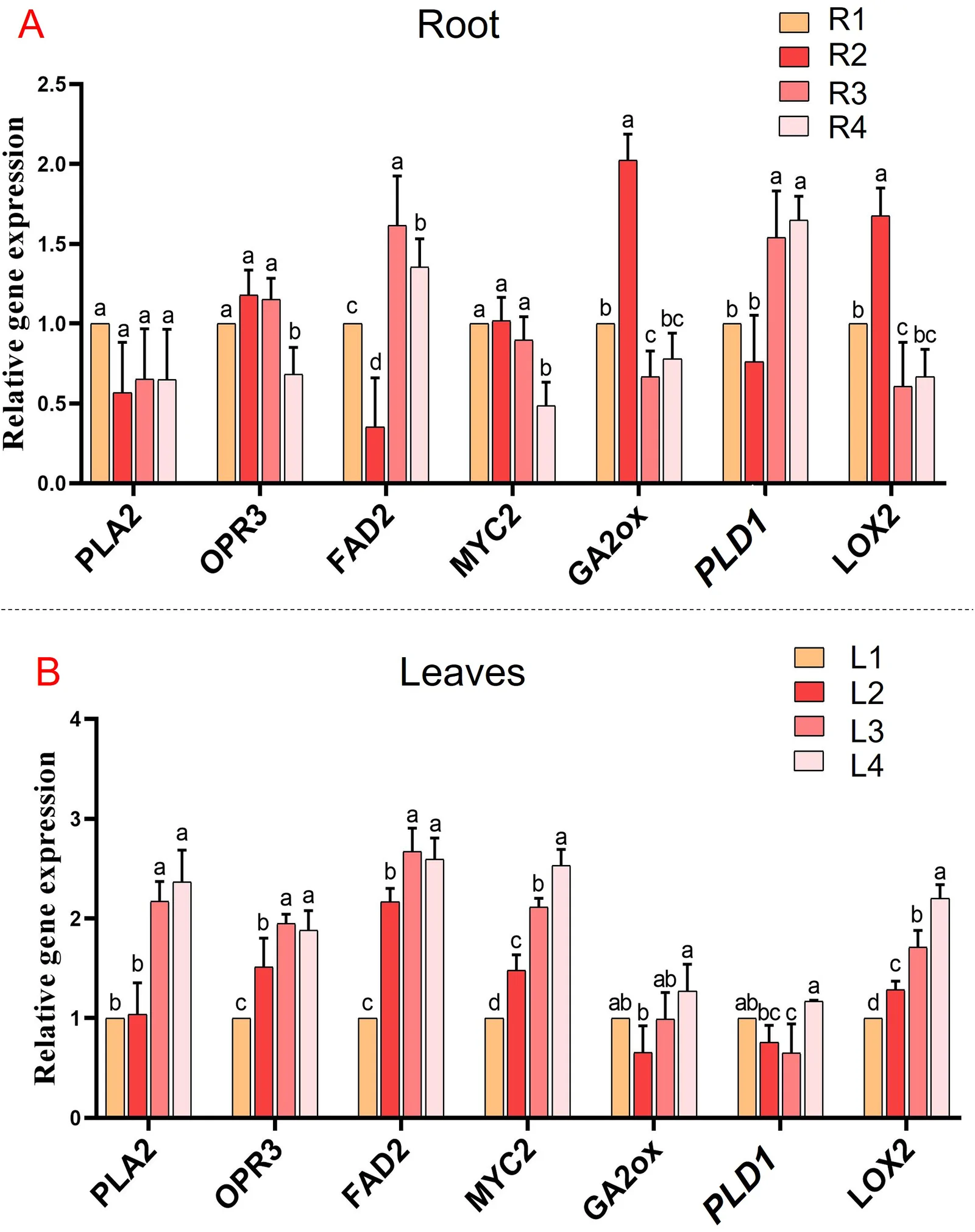
Relative expression of selected lipid-metabolism- and hormone-signaling-related genes in soybean roots and leaves. Relative expression levels of *PLA2*, *OPR3*, *FAD2*, *MYC2*, *GA20ox*, *PLD1*, and *LOX2* in **(A)** roots and **(B)** leaves. R1 and L1 represent root and leaf measurements under control conditions, respectively; R2 and L2 represent nitrogen deficiency; R3 and L3 represent heat stress (HS); and R4 and L4 represent combined nitrogen deficiency and heat stress. *PLA2*, phospholipase A2; *OPR3*, 12-oxophytodienoate reductase 3; *FAD2*, fatty acid desaturase 2; *MYC2*, MYC2 transcription factor; *GA20ox*, gibberellin 20-oxidase; *PLD1*, phospholipase D1; and *LOX2*, lipoxygenase 2. *Actin* was used as the reference gene. Bars represent means ± SE. For each gene within each tissue, different lowercase letters indicate significant differences among treatments at *p* < 0.05.

In roots, gene-expression responses were stress specific (**Figure 6A**). *FAD2* was downregulated under R2 but upregulated under R3 and R4, with the highest expression observed under R3. *LOX2* was upregulated under R2 and downregulated under R3, whereas its expression under R4 did not differ significantly from the control. *OPR3* and *MYC2* were downregulated only under R4. *PLA2* expression remained unchanged across all treatments. *PLD1* was upregulated under R3 and R4 but remained unchanged under R2. In contrast, *GA20ox* was upregulated under R2 and downregulated under R3, whereas its expression under R4 did not differ significantly from the control (**Figure 6A**).

In leaves, the jasmonate-biosynthesis-related genes *LOX2* and *OPR3* were significantly upregulated under all three stress treatments. Under L2, their expression reached 1.28- and 1.51-fold that of the control, respectively, and remained significantly elevated under L3 and L4. *FAD2* was also significantly upregulated under L2, L3, and L4. In contrast, *PLA2* was significantly upregulated under L3 and L4 but did not differ significantly from the control under L2. *MYC2* expression increased progressively across L2, L3, and L4, with the highest expression observed under L4. *GA20ox* did not differ significantly from the control under any stress treatment, although its expression was significantly higher under L4 than under L2. *PLD1* expression was significantly lower than the control under L3, whereas its expression under L2 and L4 did not differ significantly from the control (**Figure 6B**).

## 4 Discussion

Anthropogenic climate change is increasing the frequency of concurrent abiotic extremes, creating novel physiological challenges that threaten crop yields worldwide (Chaudhry and Sidhu, 2022). Our study provides a tissue-specific lipids profiling of soybean’s response to the agronomically critical combination of nitrogen deficiency and heat stress. We found the combined stress to be the most physiologically deleterious, significantly exceeding the impact of individual stresses in suppressing biomass accumulation, net photosynthetic rate (Pn), and photosynthetic pigment content (**Figure 1**). This severe growth inhibition was associated with the greatest oxidative stress, evidenced by higher H_2_O_2_, membrane lipid peroxidation (MDA) contents in both roots and leaves. These changes were accompanied by increased relative oxylipin abundance, indicating broader reprogramming of enzymatic and non-enzymatic oxidative lipid metabolism (**Figure 2**). This aligns with the established model whereby compound stresses often inflict synergistic damage by overloading shared physiological pathways (Zandalinas and Mittler, 2022). At the lipidomic level, some responses confirmed patterns previously reported under individual stresses. In particular, the decreased abundance of a feature putatively annotated as leaf MGDG under HS was consistent with established heat-induced galactolipid remodeling, while changes in root PA- and DG-related features agreed with the recognized involvement of these lipids in stress-associated membrane remodeling and signaling (Hou et al., 2016; Higashi et al., 2018). The principal contribution of the present study is the compartment-resolved comparison showing that combined stress produced distinct patterns of lipid reprogramming in roots and leaves that were not evident from either individual stress alone.

The observed growth inhibition under combined stress underscores a severe physiological trade-off where the demands for heat protection and membrane repair collide with limited nitrogen availability. Our data show that roots and leaves employ divergent lipid metabolic strategies. In roots, individual HS (R3) produced the most pronounced lipid- remodeling response. The turquoise module showed coordinated increases in features putatively annotated as PA, DGs and a GA12-related compound, reflects an investment in membrane fluidity, lipid signaling, and potentially growth reorientation to cope with thermal damage (**Table 1**). This is consistent with reports that heat stress triggers membrane lipid remodeling and activates gibberellin signaling to modulate growth (Colebrook et al., 2014; Liu et al., 2019). The upregulation of *PLD1* under R3 was directionally consistent with the increased abundance of putatively annotated PA- and DG-related features. In contrast, the putatively annotated GA12-related feature increased under R3, whereas *GA20ox* expression decreased. Thus, changes in the GA-related feature abundance and relative *GA20ox* expression were not directionally concordant (**Figure 6A**; **Table 1**). Under combined stress, *OPR3* and *MYC2* expression decreased together with a marked reduction in a feature putatively annotated as methyl jasmonate. In contrast, nitrogen deficiency alone did not significantly alter root *MYC2* expression.

In contrast, leaves showed their most pronounced lipid-remodeling response under combined stress. The depletion of photosynthetic membrane lipids, particularly MGDG and polyunsaturated DGs in the blue module, was consistent with previously reported heat-induced remodeling of chloroplast membranes (Narayanan et al., 2020) (**Table 1**). However, compared with the individual treatments, combined stress produced more extensive leaf lipid remodeling accompanied by changes in oxylipin-related features. Concurrently, the relative abundance of a feature putatively annotated as linoleic acid increased, together with changes in putatively annotated jasmonate-related features, including (−)-jasmonic acid and cis-jasmone (**Table 1**). Because polyunsaturated fatty acids are substrates for oxylipin biosynthesis, these changes suggest a possible alteration in oxylipin metabolism under combined stress (Wasternack and Feussner, 2018). This interpretation aligns with recent models of jasmonate-mediated defense coordination, in which alternative, potentially less growth-inhibitory oxylipins may help plants balance growth and defense under combined stress (Xu et al., 2026). However, the present steady-state abundance data do not demonstrate metabolic rerouting, pathway direction or metabolic flux. These lipidomic responses were accompanied by the upregulation of *FAD2*, *LOX2*, *OPR3*, and *MYC2* under the stress treatments, whereas *PLA2* was upregulated specifically under L3 and L4. *LOX2* and *OPR3* participate in jasmonate biosynthesis, while *MYC2* is a major transcriptional regulator of JA-responsive processes (Wasternack and Song, 2016). The observed expression patterns may therefore indicate coordinated regulation of fatty-acid desaturation, oxylipin biosynthesis, and JA signaling in stressed leaves, consistent with the involvement of oxylipin pathways in plant responses to combined stresses (Savchenko et al., 2014).

**Figure 7:**
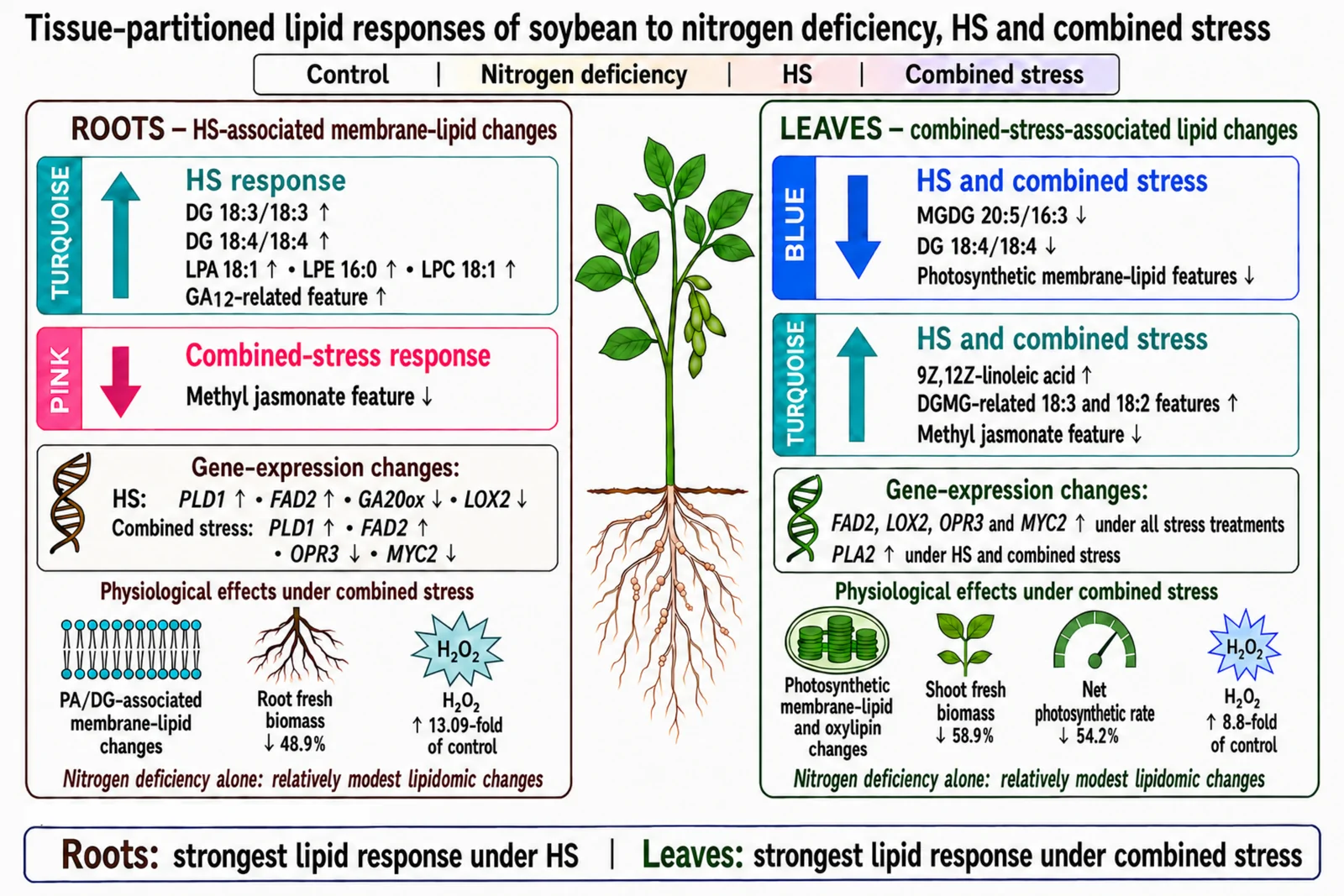
Schematic overview of tissue-partitioned lipid reprogramming in soybean under nitrogen deficiency, heat stress (HS), and their combination. Roots exhibited their strongest lipid response under HS, characterized by increased membrane-associated lipids in the turquoise module and corresponding changes in *PLD1*, *FAD2*, *GA20ox*, and *LOX2* expression. Under combined stress, methyl jasmonate decreased in the root pink module, accompanied by reduced *OPR3* and *MYC2* expression. Leaves exhibited their strongest response under combined stress, with depletion of photosynthetic membrane lipids in the blue module and increased linoleic acid and DGMG features in the turquoise module. These changes were accompanied by increased expression of *FAD2, LOX2, OPR3, MYC2*, and *PLA2*. Physiological responses under combined stress included reduced root and shoot fresh biomass and net photosynthetic rate, together with increased H_2_O_2_ accumulation. Upward and downward arrows indicate increases and decreases, respectively, relative to the corresponding Control. DG, diacylglycerol; LPA, lysophosphatidic acid; LPE, lysophosphatidylethanolamine; LPC, lysophosphatidylcholine; MGDG, monogalactosyldiacylglycerol; DGMG, digalactosylmonoacylglycerol; GA12, gibberellin A12.

Tissue-specific differences were also evident in lipid-associated metabolic and signaling responses. In roots, *PLD1* upregulation under R3 and R4 occurred alongside changes in putatively annotated PA-related features (**Figure 6A**; **Table 1**), whereas in leaves, *PLA2* upregulation under L3 and L4 coincided with changes in fatty-acid- and oxylipin-related features. (**Figure 6B**; **Table 1**). These patterns are consistent with the established involvement of PLD-derived PA in abiotic-stress signaling and phospholipase-dependent fatty-acid release in oxylipin metabolism (Hou et al., 2016; Wasternack and Feussner, 2018). Overall, roots showed PA/DG-associated membrane-lipid changes and altered GA-related features, whereas leaves showed depletion of photosynthetic membrane-lipid features and changes in oxylipin-related features. Previous studies have similarly demonstrated heat-induced chloroplast MGDG remodeling in *Arabidopsis* leaves and extensive changes in soybean leaf lipid profiles under heat stress (Higashi et al., 2018; Narayanan et al., 2020). The principal tissue-specific lipidomic, gene-expression and physiological responses observed in the present study are summarized in **Figure 7**. PLS-DA showed clear separation of lipid profiles by tissue and treatment (**Figure 3B**), whereas the separately constructed WGCNA networks identified tissue-specific lipid co-abundance modules associated with physiological traits (**Figures 5B–E**). In roots, the blue and pink modules were generally positively correlated with biomass, N content and photosynthetic traits and negatively correlated with H_2_O_2_, MDA and TOA, whereas the turquoise module showed the opposite pattern. In leaves, the blue and yellow modules were generally positively correlated with growth and photosynthetic traits and negatively correlated with H_2_O_2_, MDA and TOA, whereas the turquoise module showed broadly opposite associations. These module–trait relationships identified organ-specific lipid co-abundance patterns associated with plant physiological status. However, they do not establish the functional roles of individual modules, causal regulation, treatment-specific effects or communication between roots and leaves.

Although parallel changes occurred in roots and leaves, the present study does not demonstrate transport of specific lipids between these tissues. Targeted LC–MS/MS using authentic standards is required to confirm the identities and quantify the key PA-, DG-, JA- and GA-related features identified here. Vascular-sap profiling could establish whether these candidate compounds are present in the xylem or phloem during combined stress. Grafting experiments could determine whether root responses influence leaf oxylipin profiles, or whether shoot responses affect lipid remodeling in roots. However, grafting alone would not identify the transported molecule. Stable-isotope tracing could then distinguish locally synthesized metabolites from those transported between organs, particularly for the observed JA-related and fatty-acid features. Together, these approaches would help distinguish independent organ-specific responses from genuine root–shoot metabolic communication.

## 5 Conclusions

This study demonstrates that nitrogen deficiency, HS and their combination produce distinct tissue-dependent physiological and lipidomic responses in soybean under controlled hydroponic conditions. Combined stress caused the greatest physiological impairment and the most pronounced lipidomic shift in leaves, whereas roots showed their strongest lipidomic response under HS. Roots exhibited changes in putatively annotated PA- and DG-related features accompanied by altered *PLD1* expression, while leaves showed depletion of putatively annotated photosynthetic membrane-lipid features and changes in oxylipin-related features. These compartment-resolved patterns identify candidate lipid-associated processes for further investigation but neither demonstrate root–shoot lipid transport nor establish validated breeding targets. Future studies should combine targeted lipid and hormone quantification with vascular-sap profiling, grafting and stable-isotope tracing to determine whether specific lipids function as mobile root–shoot signals. Validation across genetically diverse soybean lines and under field conditions is also needed to determine whether these responses can inform strategies for improving resilience to combined nitrogen deficiency and heat stress.

## Data Availability

The original contributions presented in the study are included in the article/**Supplementary Material**. Further inquiries can be directed to the corresponding authors.
